# Patient-Specific iPSC-Derived Neural Differentiated and Hepatocyte-like Cells, Carrying the Compound Heterozygous Mutation p.V1023Sfs*15/p.G992R, Present the “Variant” Biochemical Phenotype of Niemann-Pick Type C1 Disease

**DOI:** 10.3390/ijms222212184

**Published:** 2021-11-10

**Authors:** Christin Völkner, Maik Liedtke, Robert Untucht, Andreas Hermann, Moritz J. Frech

**Affiliations:** 1 Translational Neurodegeneration Section “Albrecht Kossel”, Department of Neurology, University Medical Center Rostock, 18147 Rostock, Germany; Christin.Voelkner@med.uni-rostock.de (C.V.); Maik.Liedtke@med.uni-rostock.de (M.L.); andreas.hermann@med.uni-rostock.de (A.H.); 2 Department of Neurology, University Hospital Carl Gustav Carus, Technische Universität Dresden, 01307 Dresden, Germany; Robert.Untucht@uniklinikum-dresden.de; 3 Center for Transdisciplinary Neurosciences Rostock (CTNR), University Medical Center Rostock, 18147 Rostock, Germany; 4 German Center for Neurodegenerative Diseases (DZNE) Rostock/Greifswald, 18147 Rostock, Germany

**Keywords:** NP-C1, induced pluripotent stem cells, iPSCs, filipin, cholesterol, NPC1

## Abstract

Niemann–Pick disease type C1 (NP-C1) is a rare lysosomal storage disorder caused by autosomal recessive mutations in the *NPC1* gene. Patients display a wide spectrum on the clinical as well as on the molecular level, wherein a so-called “variant” biochemical phenotype can be observed. Here, we report an in vitro analysis of fibroblasts obtained from an NP-C1 patient carrying the undescribed compound heterozygous mutation p.V1023Sfs*15/p.G992R. Since NP-C1 is a neurovisceral disease and the patient suffers from severe neurological as well as hepatic symptoms, we extended our study to neural differentiated and hepatocyte-like cells derived from patient-specific induced pluripotent stem cells. We detected slightly increased intracellular cholesterol levels compared to the control cell line in fibroblasts, neural differentiated and hepatocyte-like cells, suggesting a “variant” biochemical phenotype. Furthermore, the total NPC1 protein, as well as post-ER glycoforms of the NPC1 protein, tended to be reduced. In addition, colocalization analysis revealed a mild reduction of the NPC1 protein in the lysosomes. The patient was diagnosed with NP-C1 at the age of 34 years, after an initial misdiagnosis of schizophrenia. After years of mild and unspecific symptoms, such as difficulties in coordination and concentration, symptoms progressed and the patient finally presented with ataxia, dysarthria, dysphagia, vertical supranuclear gaze palsy, and hepatosplenomegaly. Genetic testing finally pointed towards an NP-C1 diagnosis, revealing the so-far undescribed compound heterozygous mutation p.V1023Sfs*15/p.G992R in the *NPC1* gene. In light of these findings, this case provides support for the p.G992R mutation being causative for a “variant” biochemical phenotype leading to an adult-onset type of NP-C1 disease.

## 1. Introduction

Niemann–Pick disease type C1 (NP-C1) is a neurovisceral disease caused by mutations in the *NPC1* gene [1]. As the NPC1 protein is involved in the endosomal processing of cholesterol, as well as other lipids, mutations lead to the sequestration of cholesterol and glycosphingolipids in lysosomes [1]. On the clinical as well as on the molecular level, there is extensive phenotypic variability. The clinical range comprises neonatal rapidly fatal, early/late infantile, juvenile, and adolescent/adult forms [1]. In many patients, the onset of the disease is observed between the ages of 6 and 15. This so-called juvenile form of NP-C1 is the most common course of the disease. Patients suffering from the juvenile form decease between 10 and 25 years of age. Patients with a later onset, such as the adolescent and adult form with an onset later than 15 years of age, show the first neurological symptoms often in the second or third decade. These patients may present isolated psychiatric symptoms for many years before the onset of motor and cognitive abnormalities [2,3,4]. Common psychiatric symptoms are psychoses, including auditory and visual hallucinations and thought disorders. These symptoms may be acute or progressive, and patients might show relapses. Depression, behavioral symptoms, and social isolation constitute other psychiatric signs. Commonly observed symptoms are cerebellar ataxia (76%), vertical supranuclear ophthalmoplegia (75%), dysarthria (63%), cognitive impairment (61%), movement disorders (58%), psychiatric disorders (45%), dysphagia (37%), and splenomegaly (54%). Epilepsy is not very frequent in patients with an adult-onset type of NP-C1 (15%, for review see [1]).

For the laboratory diagnosis of NP-C1, the filipin test represented the gold standard for a long time. For this purpose, primary human fibroblasts are cultured and stained with the fluorescent polyene antibiotic filipin, which binds specifically to 3-beta-hydroxysterols such as free cholesterol [5]. The characteristic perinuclear light blue staining is indicative of NP-C1. However, only about 80% of patients exhibit this classic staining. Up to 20% of NP-C1 patients are diagnosed with a “variant” biochemical phenotype characterized by faint filipin staining reflecting mild abnormalities in cholesterol accumulation in lysosomes due to less-severe impairment of intracellular cholesterol transport [6,7]. The cysteine-rich luminal loop of the NPC1 protein contains approximately one-third of all described mutations, with a variable cellular and clinical impact [7]. Interestingly, mutations leading to a “variant” biochemical phenotype are typically located on this loop [7,8,9]. The “variant” biochemical phenotype is suggested to be overrepresented in patients with adult onset of NP-C1 [6,9] and tends to correlate with a less-rapid disease course. Given the wide spectrum of disease presentation that is seen in NP-C1, it is important to report patient-specific mutations, as well as phenotypic heterogeneity.

Here, we analyzed patient-specific fibroblasts, carrying the undescribed compound heterozygous mutation p.V1023Sfs*15/p.G992R, by means of filipin staining and expression, as well as localization of the NPC1 protein. Furthermore, we extended these analyses to iPSC-derived neural differentiated and hepatocyte-like cells, as NP-C1 patients suffer from neurological and systemic symptoms.

## 2. Results

### 2.1. Generation of iPSC-Derived Neural Differentiated Cells

**Figure 1:**
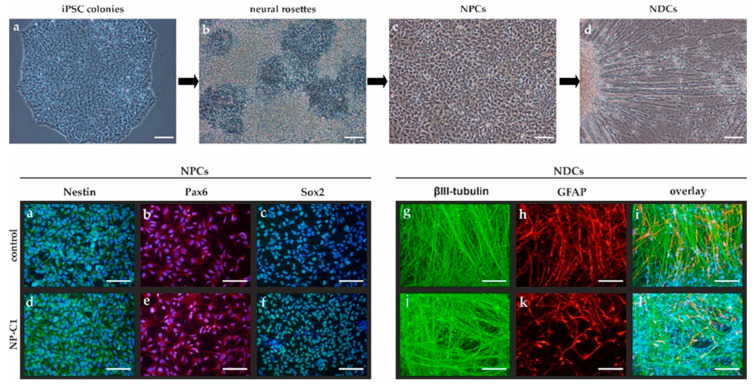
**Neural differentiation. Upper panel**: Spontaneous differentiation of iPSC colonies (**a**) was used to obtain neural rosettes (**b**) containing neural progenitor cells (NPCs, **c**). NPCs were isolated and used to obtain neural differentiated cells (NDCs, **d**). **Lower panel**: NPCs were positive for Nestin (green, **a**,**d,** DAPI in blue to counterstain for nuclei), Pax6 (red, **b**,**e**, DAPI in blue to counterstain for nuclei), and Sox2 (green, **c**,**f**, DAPI in blue to counterstain for nuclei). NPCs were terminally differentiated in NDCs positive for the neuronal marker βIII-tubulin (green, **g**,**j**) or the glial cell marker GFAP (red, **h**,**k**). (**i**,**l**) overlay of (**g**,**h**) and (**j**,**k**), respectively, and DAPI in blue to counterstain for nuclei. Scale bar = 100 µm.

Neural differentiation of iPSCs was induced as described previously by Peter and co-workers [10]. In brief, density-dependent growth of iPSC colonies (Figure 1a, upper panel) induces the spontaneous formation of neural rosettes (Figure 1b, upper panel), which were used to isolate neural progenitor cells (NPCs; Figure 1c, upper panel) using magnetic beads against the surface marker polysialylated neural cell adhesion molecule (PSA-NCAM), a marker of the neural lineage. Subsequently, NPCs were cultured over a period of 6 weeks, resulting in neural-differentiated cells (NDCs; Figure 1d, upper panel). NPCs were proven to be positive for the neural markers Nestin, Pax6, and Sox2 (Figure 1a–f, lower panel) and NDCs consist of βIII-tubulin-positive neurons (Figure 1g,i,j,l, lower panel) and glial fibrillary acidic protein (GFAP)-positive glial cells (Figure 1h,i,k,l, lower panel). 

### 2.2. Generation of iPSC-Derived Hepatocyte-like Cells

**Figure 2:**
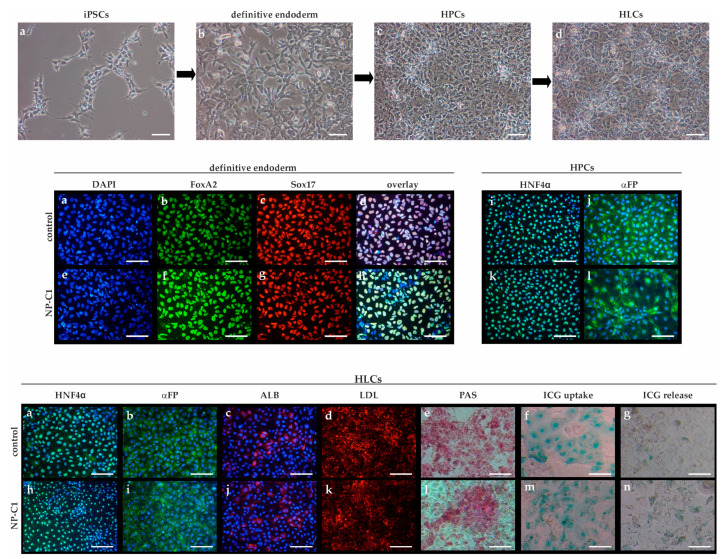
**Hepatic differentiation from hiPSCs. Upper panel**: Schematic representation of the hepatic differentiation protocol. (**a**) iPSCs, (**b**) definitive endoderm cells, (**c**) hepatic progenitor cells (HPCs), (**d**) hepatocyte-like cells (HLCs). **Middle panel**: (**a**,**e**) DAPI staining for visualization of nuclei. (**b**,**f**) Staining of FoxA2 (green) and (**c**,**g**) Sox17 (red), (**d**,**h**) overlay of (**a**–**c**), and (**e**–**g**), respectively. (**i**,**k**) Staining of HNF4α (green, DAPI in blue), (**j**,**l**) staining of alpha-fetoprotein (αFP, green, DAPI in blue). **Lower panel**: Hepatocyte-like cells (HLCs) expressed lineage-specific markers such as HNF4α (**a**,**h**, green, DAPI in blue), α-fetoprotein (αFP, **b**,**i**, green, DAPI in blue), and human albumin (ALB, **c**,**j**, red, DAPI in blue). HLCs displayed functional characteristics, such as the ability to uptake DiI-Ac-LDL (**d**,**k**, red), glycogen synthesis, and storage as shown by PAS staining (**e**,**l**) and uptake (**f**,**m**) and release (**g**,**n**) of indocyanine green (ICG). Scale bar = 100 µm.

Hepatic differentiation was performed based on a multi-step protocol of Wang and co-workers [11]. First, iPSCs (Figure 2a, upper panel) were differentiated into definitive endoderm cells (Figure 2b, upper panel), which were subsequently differentiated into hepatic progenitor cells (HPCs, (Figure 2c, upper panel) and finally into hepatocyte-like cells (HLCs, Figure 2d, upper panel). Definitive endoderm cells were positive for the markers FoxA2 and Sox17 (Figure 2a–h, middle panel). Hepatic progenitor cells (HPCs) expressed fetal hepatic markers HNF4α (Figure 2i,k, middle panel) and alpha-fetoprotein (αFP, Figure 2j,l, middle panel). Hepatocyte-like cells (HLCs, Figure 2, lower panel) showed a characteristic polygonal morphology and expressed lineage-specific markers such as HNF4α (Figure 2a,h, lower panel), α-fetoprotein (αFP, Figure 2b,i, lower panel), and human albumin (ALB, Figure 2c,j, lower panel). HLCs displayed functional characteristics of primary hepatocytes, such as the uptake of DiI-Ac-LDL (Figure 2d,k, lower panel), glycogen synthesis, and storage as demonstrated by Periodic Acid-Schiff (PAS) staining (Figure 2e,l, lower panel) and the uptake (Figure 2f,m, lower panel) and release (Figure 2g,o, lower panel) of indocyanine green (ICG).

### 2.3. Cholesterol Accumulation in Patient-Specific Fibroblasts and iPSC-Derived Neural Differentiated and Hepatocyte-like Cells

**Figure 3:**
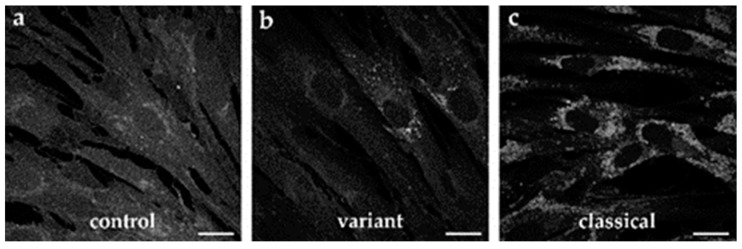
**Biochemical heterogeneity of cultured skin fibroblasts after filipin staining.** (**a**) Control fibroblasts, (**b**) the patient reported in this study showed a variant NP-C1 phenotype (V1023Sfs*15/G992R), (**c**) a classical NP-C1 mutant with compound heterozygous mutation (P543Rfs*20/E612D) is shown for comparison. Scale bar = 20 µm.

Niemann–Pick disease type C1 is generally considered a cholesterol storage disease [12]. During the diagnostic process, the accumulation of cholesterol in late endosomes and lysosomes can be shown by filipin staining of the patient′s cultured skin fibroblasts. However, biochemical heterogeneity is well known [8], including classical and variant phenotypes (Figure 3). Classical NP-C1 mutants (Figure 3c) show a massive cholesterol accumulation, which is represented by a bright fluorescent filipin staining of the fibroblasts, showing a fluorescent perinuclear pattern that is so intense that it is hardly visible as individual puncta. Variant phenotypes (Figure 3b), on the contrary, show an overall fluorescence intensity similar to control cells (Figure 3a) and the majority of cells appear to be normal, while only a small percentage of cells show weak fluorescent patterns. 

**Figure 4:**
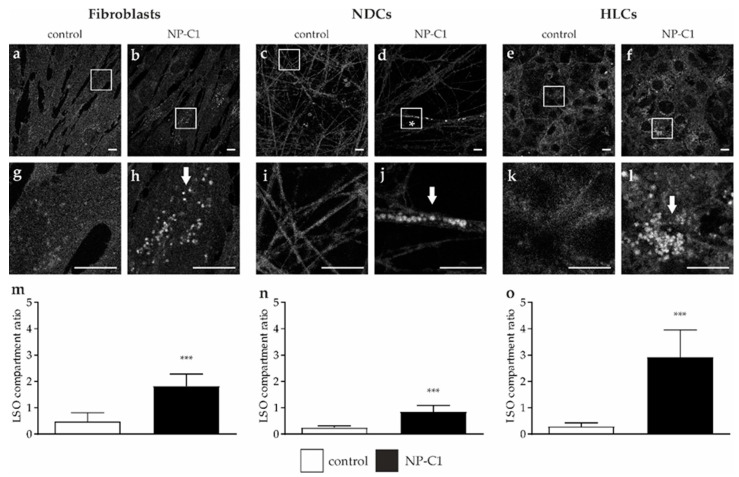
**Determination of cholesterol level.** Filipin staining was used for a qualitative analysis of cholesterol accumulations in fibroblasts (**a**,**b**), neural-differentiated cells (NDCs, **c**,**d**), and hepatocyte-like cells (HLCs, **e**,**f**). Magnified images of the squared region of each image are shown below (**g**–**l**). LSO compartment ratio was used to quantify the cholesterol accumulation and revealed a significantly increased ratio in fibroblasts (**m**), NDCs (**n**), and HLCs (**o**). Scale bar = 10 µm. *** = *p* < 0.001.

The filipin staining observed in the NPC1-deficient fibroblasts appeared weak, with an overall low level of fluorescence and only a small percentage of cells were positive for fluorescent perinuclear puncta. The same range of intensity was seen in the NDCs and HLCs, whereby the HLCs showed the highest percentage of cells with fluorescent perinuclear puncta (indicated by arrows in Figure 4b,d,f,h,j,l). The NDCs showed fluorescent puncta not only in the cell bodies but also in the extensions (marked with *, Figure 4d). The quantification of cholesterol load was performed by determining the lysosome-like storage organelle (LSO) compartment ratio [13]. Although we observed only slight differences in the fluorescence pictures, the NPC1-deficient fibroblasts showed an elevated LSO compartment ratio (1.8 ± 0.5) compared to control fibroblasts (0.5 ± 0.3, Figure 4m). Similar to the observations in the fibroblasts, NDCs (con: 0.2 ± 0.1; NP-C1: 0.8 ± 0.2) and HLCs (con: 0.2 ± 0.1; NP-C1: 2.9 ± 1.0) showed a significantly increased LSO compartment ratio, but only mild abnormalities in the filipin staining compared to control cells.

### 2.4. NPC1 Expression in Patient-Specific Fibroblasts and iPSC-Derived Neural Differentiated and Hepatocyte-like Cells

**Figure 5:**
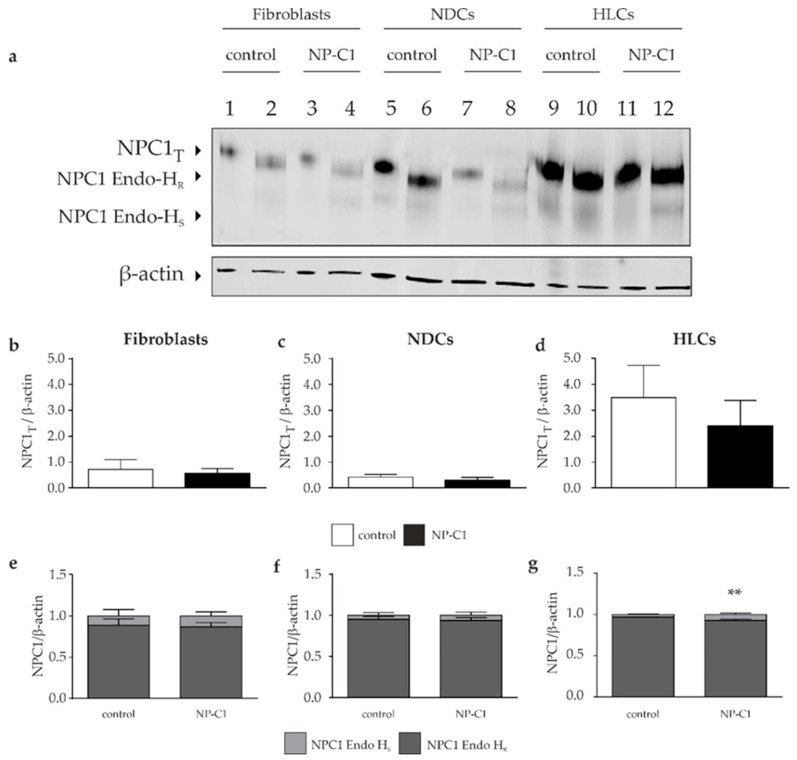
**NPC1 protein level in fibroblasts, NDCs, and HLCs.** (**a**) Western blot analysis was performed to determine the total NPC1 protein amount indicated by NPC1_T_ in lanes 1, 3, 5, 7, 9, and 11. Digestion of the samples with endoglycosidase H (Endo-H) was used to quantify the amount of Endo-H-resistant and Endo-H-sensitive amount of NPC1 protein (lane 2, 4, 6, 8, 10, and 12). (**b**–**d**) Quantification of the total NPC1 amount revealed comparable protein content in control and NP-C1 mutated cells, in the respective cell types. At the same time, the HLCs displayed more NPC1 protein than the fibroblasts or NDCs. (**e**–**g**) Irrespectively of the cell type, digestion of the samples with endoglycosidase H revealed comparable high amounts of Endo-H-resistant NPC1 protein portion. ** = *p* < 0.01.

To assess the expression level of the total NPC1 protein, we performed Western blotting of cell lysates of fibroblasts, neural-differentiated, and hepatocyte-like cells (Figure 5). We found only slightly reduced, but not significantly different, amounts of total NPC1 protein levels. However, we observed an up to 8-times higher NPC1 amount in HLCs, in comparison to the fibroblasts and NDCs.

To analyze the portion of the NPC1 protein proned for degradation, due to the mutation, we used endoglycosidase H for the digestion of total cell lysates, leading to the removal of immature N-linked glycan residues. The analysis by means of Western blot revealed that the majority of the NPC1 protein is present as the Endo H-resistant form in all cell types (fibroblasts con: 88 ± 7%, NP-C1: 86 ± 5%; NDCs con: 95 ± 3%, NP-C1: 93 ± 3%; HLCs 96 ± 0.4%; NP-C1: 92 ± 1%), suggesting that the protein is properly folded and trafficked through the secretory pathway. 

**Figure 6:**
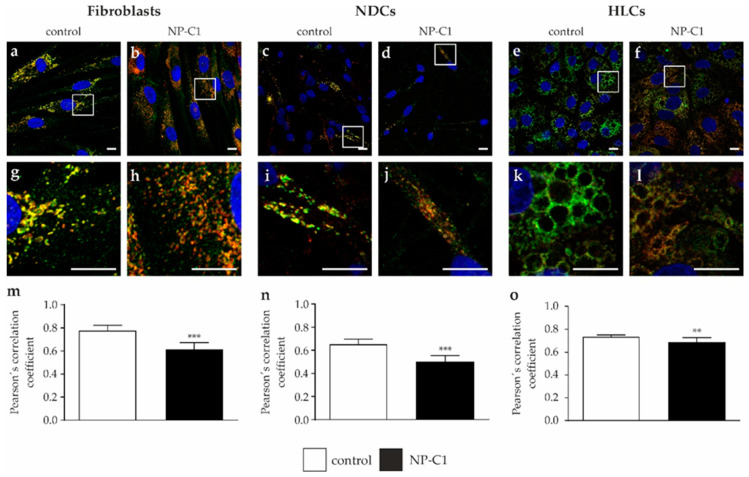
**Analysis of NPC1 localization.** Immunofluorescence pictures were used to determine the colocalization of NPC1 protein (green) and LAMP2 (red) as a lysosomal marker protein, in fibroblasts (**a**,**b**), NDCs (**c**,**d**), and HLCs (**e**,**f**). Magnified images of the squared region of each image are shown below (**g**–**l**). DAPI was used to counterstain nuclei (blue, **a**–**l**). (**m**–**o**) Calculation of Pearson’s correlation coefficient revealed strong colocalization in all cell types, although NP-C1 mutated cells showed a significantly lower colocalization. Scale bar = 10 µm. ** = *p* < 0.01, *** = *p* < 0.001.

Accordingly, we determined a large portion of the NPC1 protein in lysosomal compartments, detected by a colocalization analysis of immunofluorescence pictures of NPC1 and LAMP2 (Figure 6). The calculation of Pearson’s correlation coefficient was used to determine the colocalization of the NPC1 protein and lysosomal marker LAMP2. Although NP-C1 mutated cells showed a significantly lower localization of NPC1 in lysosomes, the results support the assumption that the mutant NPC1 protein is nevertheless transported to lysosomes to a large extent.

### 2.5. Clinical Representation

The patient presented here is a woman who apparently enjoyed good health and was symptomatically unremarkable until her early twenties. Retrospectively, the parents reported that the patient was already conspicuous for clumsiness and frequent falls as an infant. School attendance had been regular including average performance in physical education classes, and the patient successfully completed an apprenticeship. However, at the age of 24, psychosis manifested itself, which prevented further vocational training. The patient had auditory hallucinations, was depressed and listless, and became withdrawn. At the same time, a balance disorder and slurred speech were noted. This was followed by an inpatient stay in the psychiatric ward and drug treatment with a diagnosis of schizophrenia. Since then, several psychotic episodes followed, requiring repeated hospitalizations. At age 33, ataxia, dysarthria, dysphagia, and—most remarkably—vertical gaze palsy, and hepatosplenomegaly were noted, which eventually led to the tentative diagnosis of Niemann–Pick disease type C1. Interestingly, the investigation of fibroblasts from a skin biopsy in a clinical laboratory did not confirm the diagnosis at that time. At the age of 34, her symptoms worsened. Soon the patient was unable to stand and walk unassisted, she developed dystonia, spasticity and anarthria. Since the clinical syndrome fitted that well, genetic testing was performed and the diagnosis of Niemann–Pick disease type C1 was very likely due to a compound heterozygous mutation of the NPC1 gene, consisting of a frameshift (p.V1023Sfs*15) and a missense mutation (p.G992R), respectively. Taken together with the findings presented in this study, we conclude that the patient displays the “variant” biochemical phenotype, accompanied with late disease onset and rapid deterioration of the state of health, finally supporting the novel mutation described herein as a causative mutation for a variant phenotype of NP-C1. 

After genetic confirmation of the diagnosis, treatment with miglustat was started. The current treatment regimen consists of the administration of 200 mg of miglustat three times daily. At the current age of 43 years, the patient is unable to walk, get dressed, or eat by herself. Only very simple active movements are possible. She has severe deformities of most joints of both upper extremities (e.g., wrists in 100° flexed position), as well as of the legs and neck to a lesser extent. This is from dystonia more than spasticity, but there are also significant contractures. Overall, the patient can articulate only single words and short sentences but would sing songs. Attentiveness, perception, and comprehension seem to be quite good.

**Figure 7:**
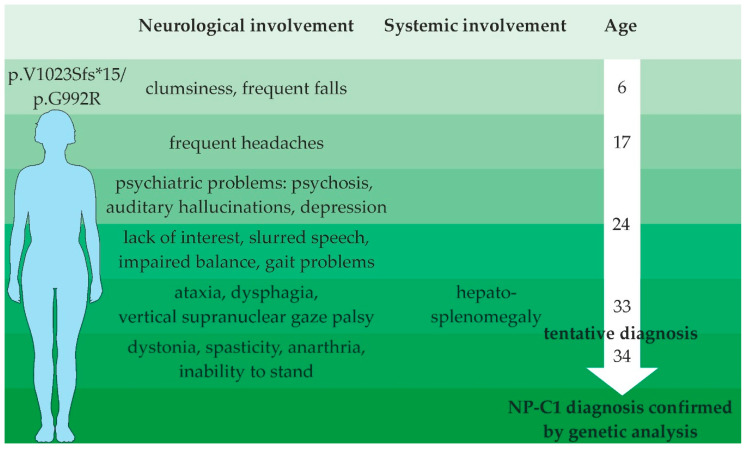
Schematic overview of the patient’s disease course.

Symptomatic treatment with physiotherapy, aquatic physical therapy, occupational therapy, and quarterly injections of botulinum toxin are implemented to improve motor function. Botulinum toxin leads to markedly improved passive movement ability, facilitating care procedures during its duration of action, though high doses are needed (currently 600–700 units of onabotulinumtoxin A for 12–14 arm muscles). An overview of the patient’s disease course is given in Figure 7 and an overview of laboratory diagnostics is presented in Table 1.

## 3. Discussion

NP-C1 is associated with a broad spectrum of clinical manifestations, at least some of which are associated with specific types of mutations. The so-called “variant” biochemical phenotype can be observed in about 20% of NP-C1 patients. This phenotype is characterized by a weak filipin staining, reflecting mild abnormalities in cholesterol accumulation in lysosomes and near-normal cholesterol esterification rates due to a less-severe impairment of intracellular cholesterol trafficking [6,14]. These patients usually present with late disease onset. It is not exactly known whether the “variant” biochemical phenotype is based solely on specific *NPC1* mutations or whether other aspects of the patient′s genetic background play a role [8]. However, there are a number of point mutations that are frequently associated with a “variant” biochemical phenotype. One example is the second-most common *NPC1* mutation in Europe, p.P1007A [7,15,16]. Even though P1007A is strongly correlated with a “variant” biochemical phenotype, Sun and colleagues found this mutation in both variant and classical samples, suggesting that interactions between both alleles play a role in determining the phenotypic type [8]. Other mutations correlating with a “variant” biochemical phenotype are D948N, S1004L, R978C, R958Q, G992W, and G992R [8], wherein the mutation p.G992R has been previously reported to be associated with late onset of the NP-C1 disease [7]. In the present study, we report data on fibroblasts, obtained from a female patient that has a compound heterozygous mutation harboring the point mutation p.G992R (c.2974G>C) on one allele in conjunction with a frameshift mutation p.V1023Sfs*15 (c.3066_3073delinsT) on the other allele, which leads to premature protein truncation. In addition, we analyzed iPSC-derived cells in regards of the presentation of a “variant” biochemical phenotype. To the best of the authors′ knowledge, the mutation p.V1023Sfs*15 has not yet been described, as well as, consequently, the combination with the p.G992R mutation.

### 3.1. In Vitro Analysis of Patient-Specific Fibroblasts Revealed a “Variant” Biochemical Phenotype

First, we were interested in the intracellular cholesterol level of the patient′s fibroblasts, as well as the NPC1 protein level and localization. Upon investigating the NP-C1 patient′s fibroblasts by means of filipin staining, we observed that only a small fraction of cells showed typical perinuclear puncta indicative of cholesterol accumulation, while the majority of cells had a normal appearance. This is in accordance with other studies describing a “variant” biochemical phenotype [17]. The quantification of the cholesterol accumulation by calculating the LSO compartment ratio [13] revealed a four-times-higher ratio in the NPC1 patient’s fibroblasts. However, to our experience, this level of cholesterol accumulation is rather small compared with fibroblasts from NP-C1 patients, which show a classical staining pattern. Such weak filipin staining of a “variant” biochemical phenotype can be easily missed during diagnostic testing, resulting in a delay in the diagnosis of NP-C1. However, the low level of cholesterol accumulation that we observed in the cells suggests that, despite the mutations, there is still enough functional NPC1 protein to guarantee the proper transport of cholesterol. To support this assumption, we performed a Western blot analysis. The analysis of the NPC1 protein level by Western blot revealed only a slight reduction of the total NPC1 protein, which is consistent with a recent report that suggests that a high level, of even mutated, NPC1 protein can lead to a less-severe clinical phenotype [18]. As a side note, the NPC1 antibody used here binds to the c-terminal domain of the NPC1 protein, so that a truncated protein, resulting from the transcription frameshift mutation, is not detected during analysis. However, to determine the intracellular localization of the NPC1 protein, we performed Endo-H digestion of the cell lysates to analyze NPC1 trafficking. Interestingly, digestion with Endo-H showed comparable amounts of Endo-H-resistant protein in NP-C1 cells and control cells, indicating that the majority of the protein had advanced beyond the endoplasmic reticulum (ER) in the secretory pathway. This result suggests that the mutant NPC1 protein forms a stable conformation, preventing an ER-mediated degradation of the protein. This assumption was supported by the results of colocalization analysis of the NPC1 protein and LAMP2, a lysosomal marker protein. Although the analysis revealed a significantly lower Pearson′s correlation coefficient in the NPC1 mutant fibroblasts, the calculated coefficient of 0.62 can be considered to indicate high colocalization and thus the proper transport and localization of the mutated NPC1 protein in lysosomes. A filipin staining alone might not be sufficient to identify a “variant” biochemical NP-C1 phenotype. However, the combination of a filipin assay, Western blot analysis, and colocalization analysis displayed a clear picture of a phenotype intermediate between control and mutant cell lines. The low level of cholesterol accumulation observed, but the high level of the NPC1 protein, with the majority being mature and localized correctly in late endosome/lysosomes, are in accordance with the findings of Millat et al., describing that the G992R mutation in homozygosity and in combination with another frameshift mutation leads to a “variant” biochemical phenotype [7]. Thus, we conclude that the here-described compound heterozygous mutation can be classified as a “variant” biochemical phenotype.

### 3.2. iPSC-Derived Neural Differentiated Cell, Hepatocyte-like Cells and Fibroblasts Show a Comparable Phenotype

NP-C1 disease is represented as a primarily neurodegenerative disorder; still, hepatosplenomegaly is present in nearly 50% of NP-C1 patients, regularly displaying one of the first detectable symptoms [1]. Patients with the neonatal rapidly fatal form of NP-C1 usually die from liver failure before the age of 6 months [19,20]. However, NP-C1 deficiency in those human disease-affected cell types has only been investigated to a limited extent because of a lack of availability of appropriate cell material. Thus, less is known about the molecular NP-C1 phenotype in different disease-affected cell types. Recently, we described the generation of NP-C1 patient-specific iPSCs, derived from fibroblasts of the patient described here [21]. We used these iPSCs to generate neural differentiated and hepatocyte-like cells and subjected them to the same analyses as the patient-specific fibroblasts.

Regarding the analysis of cholesterol accumulation by filipin staining, we observed cholesterol accumulation in NDCs and HLCs. The observation of cholesterol accumulation in NDCs is in accordance with our previous findings in NPC1-deficient NDCs [22,23], as well as with findings of other studies [24]. Regarding the accumulation of cholesterol in hepatocyte-like cells, only two other studies reported on this NP-C1 specific disease hallmark [24,25]. In accordance with these studies, we observed cholesterol accumulation in HLCs. However, a comparison to the other studies is hardly feasible, as the analyzed NPC1-deficient cells carry other mutations. However, the cellular phenotype we observed in our NDCs and HLCs is comparable with the phenotype of the patient-specific fibroblasts, reflecting a “variant” biochemical phenotype. The same holds true for the analysis of the total NPC1 protein amount and the Endo-H-resistant protein amount. Although the three different cell types analyzed did not differ significantly between the NPC1 mutated cells and the control cells, we observed an increased amount of the NPC1 protein in HLCs compared to fibroblasts and NDCs. 

Taken together, our data show that patient-specific iPSC-derived neural-differentiated and hepatocyte-like cells show the same disease-relevant defects as the corresponding primary fibroblasts, such as defective cholesterol metabolism and changes in the NPC1 protein level and maturation. Thus, we conclude that the iPSC-derived cell systems reflect a “variant” biochemical, comparable to the phenotype of the patient-specific fibroblasts, confirming the feasibility of the iPSC-based model system to model the NP-C1 phenotype and, moreover, to serve as a basis for further studies to elucidate pathophysiological mechanisms of NP-C1 in disease-related tissues.

### 3.3. Cinical Presentation of a Patient with a “Variant” Biochemical Phenotype

The clinical presentation of a “variant” biochemical phenotype is often associated with an adult-onset, slowly progressive disease course but still leading to severe symptoms. These patients often show the first neurological symptoms in the second or third decade and may present isolated psychiatric symptoms for several years before the onset of motor and cognitive impairments. The here-presented case presents a typical disease progression of an adult-onset (>15 years) patient. The disease first manifested itself by psychiatric symptoms, which were then followed by typical neurological and motor symptoms. Systemic symptoms, such as hepatosplenomegaly, are present in more than half of the patients with an adult disease onset. Accordingly, the patient showed liver and spleen enlargement. Regarding laboratory diagnostics, several studies on NP-C1 reported deviations from reference values. Tängemeo and co-workers investigated the plasma levels of HDL-cholesterol in NP-C1 patients and found that they are decreased in patients with the classical phenotype while they tend to be in the normal range in patients with a variant phenotype [17]. This also applies to the patient described in the present study, who showed an HDL-cholesterol value of 40 mg/dL (reference > 40 mg/dL) at the time of diagnosis. Low HDL-cholesterol levels in NP-C1 patients have been explained by an impaired formation of HDL particles owing to defective upregulation of ABCA1, when NPC1-deficient cells are exposed to LDL [26]. Tängemeo et al. [17] therefore hypothesized that due to residual activity of the NPC1 protein, in lysosomes of patients showing a “variant” biochemical phenotype, the regulation of ABCA1 is maintained, leading to an increase in HDL-cholesterol. Indeed, the impairment in the upregulation of ABCA1 when exposed to LDL was less pronounced in fibroblasts of variant patients compared to patients with the classical phenotype.

At the time of diagnosis and after remeasurement 9 years later, the patient showed an increased value of chitotriosidase of 203 and 353 nmol/mL/h, respectively. Chitotriosidase (ChT) is a chitinase produced by activated macrophages and its activity in serum is significantly increased in patients with lysosomal storage diseases. Ries et al. reported a mean plasma ChT activity of 856 ± 721 nmol/mL/h in NP-C patients, while a value of ≥200 nmol/mL/h is considered pathological [27]. The chitotriosidase assay has been proposed as a screening test for NP-C [28]. While chitotriosidase activity is increased in most NP-C patients, elevated ChT activities are also observed in other lysosomal storage diseases such as Gaucher disease, Niemann–Pick A and B disease, and acid lipase deficiency, and is therefore not specific [29,30]. Moreover, increased ChT activity has been reported for NP-C1 receiving miglustat treatment [30]. However, nothing is known about the differences of ChT activity between patients with classical or “variant” biochemical phenotypes. Pineda and colleagues described lower plasma ChT activities in a juvenile patient subgroup compared to early/late infantile subgroups, suggesting a less distinct elevation of ChT activity in patients with late onset [31].

Patients with adult onset die at a mean age of 38 ± 10 years [14]. At the time of this publication, the patient is 43 years old. The unusual longevity is possibly supported by the mutation leading to the observed “variant” biochemical NP-C1 disease. The underlying mutation leading to G992R is a point mutation where guanine is replaced by cytosine in the DNA sequence at position 2974. This nucleotide position is highly polymorphic with different substitutions reported [32]. The G992R mutation has been shown in homozygosity and in combination with different point and frameshift mutations [7,16,32,33,34,35,36,37]. One patient carrying the G992R mutation in homozygosity showed isolated splenomegaly but did not show any neurological signs still at age 66 years, supporting the idea of G992R leading to a mild deficit in NPC1 function [7]. The G992R in combination with the prevalent mutation I1061T was described in a female patient who showed the first neuropsychiatric symptoms at the age of 57 years (ataxia, dysphagia, dysarthria) and the first visceral symptoms at the age of 64 years (splenomegaly) and was still living at the last follow up at the age of 70 years [38]. These reports are in accordance with the assumed moderate effect of the referred mutation. The G992R mutation has generally been associated with a variant phenotype, irrespective of the second allele [7]. However, two cases are described, where G992R in combination with Q921P [32] or Y825C [16] leads to a classical biochemical phenotype, suggesting that other modifying factors possibly play a role in disease manifestation. At least for the current case, we could clearly describe the G992R mutation in combination with p.V1023Sfs*15 as a “variant” biochemical phenotype. Considering that p.V1023Sfs*15 alteration causes a loss of the C-terminus, which has been demonstrated to be necessary for proper routing and function of the NPC1 protein [39], we strongly support the idea of G992R substitution as a mutation associated with mild alterations of cholesterol transport.

### 3.4. Therapeutic Options for Patients with a “Variant” Biochemical Phenotype

In light of these findings clearly representing a “variant” biochemical phenotype, showing only the mildest abnormalities in cholesterol metabolism and maturation of NPC1 protein, the question arises: How does this influence the therapeutic options of the patient?

Currently, it is generally accepted that lipid storage is causative for subsequent neurodegeneration. However, even though NP-C1 is primarily described as a cholesterol storage disorder, the offending metabolite in NP-C1 is still the subject of intense discussion [40]. The storage material is highly complex, including different classes of lipids, such as cholesterol, sphingomyelin, glycosphingolipids, and sphingosine, each showing different patterns of accumulation in different tissues [41]. Moreover, the relationship between impaired lipid storage and neurodegeneration as well as the precise function of NPC1 and NPC2, besides cholesterol distribution, remains unclear. Increasing evidence suggests that the NPC1 protein is involved in other cellular processes, such as copper metabolism and vitamin E status, the suppression of oxidative stress, and as a cofactor of STING-mediated immune response [42,43,44].

Since 2009, miglustat (Zavesca^®^) has been approved by the European Medicines Agency for the treatment of NP-C1. Miglustat is a small iminosugar, reversibly inhibiting the glycosylceramide synthase (GCS). GCS catalyzes the first step in glycosphingolipid synthesis and thus an inhibition leads to a reduction of glycosphingolipids in the lysosomes. Miglustat is able to cross the blood–brain barrier, and when orally administered it is reported to slow down the progression of neurological symptoms of NP-C1 [45]. The positive effect of miglustat, which does not directly influence cholesterol metabolism, indicates that glycosphingolipid accumulation, rather than cholesterol storage, might be the primary pathogenetic event in NP-C1, suggesting that patients with a “variant” biochemical phenotype could benefit from treatment with miglustat. Jamrozik and colleagues [46] presented a 21-year-old male with progressive ataxia recognized at the age of 18, hepatosplenomegaly, increased levels of chitotriosidase (525 nmol/mL/h), and mild accumulation of cholesterol in cultured fibroblasts. Daily administration of miglustat improved coordination movements, gait, dysphagia, and speech, and normalization of mood disorders was observed.

Another study reported a 33-year-old male with a “variant” biochemical phenotype, showing motor difficulties, gait ataxia, dysarthria, dysphagia, vertical supranuclear gaze palsy, and splenomegaly. A treatment regime with an initial treatment of 200 mg miglustat every 8 h resulted in an improvement in the NPC Disability Scale from 11 to 7 within two years [47].

In general, based on clinical experience to date, it can take 6–12 months to observe clinical benefits in early-infantile onset cases and over 2 years in later-onset disease [48]. Against this background, the best possible patient monitoring is also important in order to decide on the use of disease-modifying therapies, in the sense of individualized therapy. As mentioned above, for patients showing a “variant” biochemical phenotype, the administration of miglustat can be expected to have a positive influence on the course of the disease.

## 4. Materials and Methods

### 4.1. Cell Culture of Human Dermal Fibroblasts

Human dermal fibroblast cell lines of an apparently unaffected individual and of the NP-C1 patient were obtained from the University of Dresden and approved by the Ethical Committee (EK45022009) of the Technische Universität Dresden, Germany [21]. Cells were cultured in Dulbecco′s modified Eagle medium (DMEM) containing high glucose supplemented with 10% fetal bovine serum and 1% penicillin-streptomycin (all Thermo Fisher Scientific, Waltham, MA, USA). Cells were seeded at a density of 30,000 cells/cm^2^. Before being seeded on coverslips, coverslips were coated with 0.1% gelatin for 30 min at 37 °C.

### 4.2. Neural Differentiation

Neural differentiation of iPSCs was induced by density-dependent growth of the iPSCs on matrigel (Corning, Corning, NY, USA). After neural rosettes had been formed spontaneously, cells were washed with DMEM/F12 (Thermo Fisher Scientific, Waltham, MA, USA), singled using Accutase^®^ (Sigma-Aldrich, St. Louis, MO, USA), and then magnetically sorted using magnetic beads against the surface marker PSA-NCAM (Miltenyi Biotec, Bergisch Gladbach, Germany), which is a marker of the neural lineage. The generated neural progenitor cells were used for 20 passages and seeded at an expansion density of 100,000 cells/cm^2^ on poly-L-ornithine (15µg/mL; Sigma-Aldrich, St. Louis, MO, USA)/laminin (10 µg/mL, Bio-Techne Corporation, Minneapolis, MN, USA)-coated dishes in a proliferation medium containing 60% DMEM/F-12, 40% DMEM, 1X B27, 0.5% penicillin-streptomycin (all Thermo Fisher Scientific, Waltham, MA, USA), 20 ng/mL FGF2 (Amsbio, Abingdon, UK), and 20 ng/mL EGF (Peprotech, Hamburg, Germany). For terminal neural differentiation, cells were plated at a density of 45,000 cells/cm^2^ in the differentiation medium, containing 60% DMEM/F-12, 40% DMEM, 1X B27, and 0.5% penicillin-streptomycin, which was changed every 4 days over a period of 6 weeks.

### 4.3. Hepatic Differentiation

Eighty percent confluent iPSC cultures were washed with DMEM/F-12, singled with the Gentle Cell Dissociation Reagent (STEMCELL Technologies, Köln, Germany) for 7 min at 37 °C and seeded onto matrigel-coated dishes at a density of 45,000 cells/cm^2^ in the mTeSR1™ medium supplemented with 10 µM Y-27632 (both STEMCELL Technologies, Köln, Germany). The next day (day 1), the medium was replaced by the endoderm-priming medium (RPMI1640, 2% B27 without vitamin A, 1% penicillin/streptomycin (all Thermo Fisher Scientific, Waltham, MA, USA) supplemented with 100 ng/mL Activin A (Peprotech, Hamburg, Germany), and 50 ng/mL Wnt3a (R&D Systems, Minneapolis, MN, USA)). The endoderm-priming medium was changed every 24 h. At day 4, the medium was changed to the KSR/DMSO differentiation medium (80% Knockout DMEM (KO-DMEM), 20% Knockout serum replacement (KSR), 0.5% GlutaMAX, 1% non-essential amino acids (NEAA), 0.1 mM beta-mercaptoethanol (all Thermo Fisher Scientific, Waltham, MA, USA), 1% DMSO (Sigma-Aldrich), 1% penicillin/streptomycin) and the medium was refreshed every 24 h. At day 9, hepatic maturation was induced by switching to the HepatoZYME maturation medium (HepatoZYME basal medium, 1% GlutaMAX, 1% penicillin/streptomycin (all Thermo Fisher Scientific, Waltham, MA, USA), 10 µM hydrocortisone 21-hemisuccinate sodium salt (HCC, Sigma-Aldrich, St. Louis, MO, USA) supplemented with 10 ng/mL HGF and 20 ng/mL OSM (both Peprotech, Hamburg, Germany)). The medium was replaced every other day until day 17.

### 4.4. Periodic Acid-Schiff Assay

Glycogen storage of hepatocyte-like cells was evaluated by performing PAS staining at day 17 of differentiation. Cells were fixed with 4% PFA for 10 min at room temperature and stained using a periodic acid-Schiff (PAS) staining system (Sigma-Aldrich, St. Louis, MO, USA) according to the manufacturer′s instructions.

### 4.5. Cellular Uptake and Release of Indocyanine Green

Indocyanine green (ICG, Sigma-Aldrich, St. Louis, MO, USA) was solved in DMSO for a stock of 5 mg/mL and freshly diluted in the HepatoZYME maturation medium to 1 mg/mL. Differentiated hepatocyte-like cells at day 17 were incubated in diluted ICG for 30 min at 37 °C. After washing, the cellular uptake of ICG was documented by light microscopy. Cells were returned to the culture medium and incubated for 6 h. Subsequently, the release of cellular ICG was documented.

### 4.6. Uptake of Low-Density Lipoprotein (LDL)

The DiI-Ac-LDL staining kit was purchased from Cell Applications, Inc. and the assay was performed according to the manufacturer′s instructions. After LDL uptake, cells were mounted with Fluoromount-G^®^ (SouthernBiotech, Birmingham, AL, USA) and visualized using a Biozero 8000 microscope system (Keyence, Osaka, Japan).

### 4.7. Filipin Staining

Cells were grown on glass cover slips. Neural differentiated and hepatocyte-like cells were loaded with 60 µg/mL LDL (Sigma-Aldrich, St. Louis, MO, USA) for 48 h prior to filipin staining. Cells were fixed with 4% PFA in PBS at pH 7.4 for 10 min at room temperature and stained with 0.1 mg/mL filipin (Polysciences Europe GmbH, Hirschberg an der Bergstrasse, Germany) for 45 min in the dark. After washing with PBS, the coverslips were mounted with Fluoromount-G^®^ (SouthernBiotech, Birmingham, AL, USA). Images were captured on a Laser Scanning Microscope 900 (Zeiss) using ZEN software. The LSM 900 was equipped with a motorized scanning stage 130 × 100 STEP, a laser module URGB with a 405 nm diode laser, a Plan-APOCHROMAT 63x/1.4 Oil objective, and a GaAsP-PMT detector. Quantitative analysis of filipin images from 10 fields of cells/experiment was performed using ImageJ software (NIH, Bethesda, MD, USA), following the ‘LSO compartment ratio assay’ [13].

### 4.8. Immunocytochemistry

Cells were fixed at room temperature for 10 min in 4% PFA in PBS at pH 7.4, washed with PBS with calcium and magnesium (PBS^+/+^), and stored in 0.02% NaN_3_ at 4 °C. Immunocytochemistry was performed for Nestin (1:50, rabbit IgG, abcam, Cambridge, United Kingdom), Pax6 (1:100, rabbit IgG, abcam, Cambridge, United Kingdom), Sox2 (1:100, rabbit IgG, abcam, Cambridge, United Kingdom), βIII-tubulin (1:100, mouse IgG, Santa Cruz, Dallas, TX, USA), GFAP (1:500, rabbit IgG, Agilent Technologies, Santa Clara, CA, USA), FoxA2 (1:200, rabbit IgG, Cell Signaling Technology Europe, Frankfurt am Main, Germany), Sox17 (1:100, goat IgG, R&D Systems, Minneapolis, MN, USA), HNFα (1:50, mouse IgG, Santa Cruz), α-FP (1:250, mouse IgG, Sigma-Aldrich, St. Louis, MO, USA), and albumin (1:100, goat IgG, Thermo Fisher Scientific, Waltham, MA, USA). Cells were permeabilized using 0.1% Triton-X-100 in PBS for 5 min on ice and subsequently blocked with 1% BSA for 30 min at room temperature, followed by incubation with primary antibodies in 1% BSA at 4 °C overnight. The next day, cells were washed with 1% BSA, followed by incubation with secondary antibodies. Alexa Fluor 488 (1:500, goat anti-rabbit IgG or goat anti-mouse IgG), Alexa Fluor 568 (1:500, goat anti-rabbit IgG) (all Thermo Fisher Scientific, Waltham, MA, USA), or CY3 (1:400, donkey anti-goat IgG) (Jackson ImmunoResearch, Cambridgeshire, United Kingdom) were used as secondary antibodies, incubated for 2 h at room temperature in 1% BSA. After washing with PBS, cells were stained with DAPI (250 ng/mL, 5 min), washed three times with PBS, and mounted with Fluoromount-G^®^ (SouthernBiotech, Birmingham, AL, USA). Pictures were taken with a Biozero 8000 microscope system (Keyence, Osaka, Japan).

### 4.9. Colocalization Analysis

Cells grown on coverslips were washed with phosphate buffered saline (PBS) with calcium and magnesium (PBS^+/+^) and fixed with ice-cold acetone for 5 min. Cells were subsequently washed with PBS^+/+^ and incubated with permeabilization buffer containing 0.1% Triton X-100 in PBS for 5 min on ice. After a washing step with PBS^+/+^, cells were blocked with 1% BSA for 30 min at room temperature, followed by incubation with primary antibodies in 1% BSA at 4 °C overnight. The antibodies used were rabbit anti-NPC1 (abcam, 1:1000) and mouse anti-LAMP2 (abcam, 1:50). Next, the cells were washed with 1% BSA, followed by incubation with anti-rabbit IgG conjugated with Alexa488 and anti-mouse IgG conjugated with the Alexa568 secondary antibody (Invitrogen, Waltham, MA, USA) diluted 1:1000 for 2 h at room temperature. After washing with PBS^+/+^, cells were stained with DAPI (250 ng/mL, 5 min). Cover slips were mounted using Fluoromount-G^®^ (SouthernBiotech, Birmingham, AL, USA). Microscopy was performed using a Laser Scanning Microscope 900 (Zeiss) and ZEN software. The Laser Scanning Microscope 900 was equipped with a motorized scanning stage 130 × 100 STEP, a laser module URGB with 405 nm, 488 nm, and 561 nm diode lasers, a Plan-APOCHROMAT 63×/1.4 Oil objective, and a GaAsP-PMT detector. Colocalization analysis was performed using NIH ImageJ software and the plugin JaCoP to determine Pearson′s correlation coefficient.

### 4.10. Western Blot Analysis

Cells were harvested by aspirating the medium and washing with PBS and were then lysed in RIPA buffer containing a complete protease inhibitor cocktail (Roche Diagnostics) for 25 min on ice. Insoluble cell debris was pelleted (15,000× *g* at 4 °C, 25 min), and the total protein concentration in the supernatant was determined using the Pierce BCA Protein Assay Kit (Thermo Fisher Scientific, Waltham, MA, USA). For Western blotting, 20 µg of non-boiled protein samples (37 °C, 5 min) were mixed with 5× Laemmli buffer and separated by SDS-PAGE with Criterion™ TGX precast polyacrylamide gels (4–15%, Bio-Rad Laboratories GmbH, Feldkirchen, Germany) and run for 5 min at 100 V and 20 min at 300 V. Precision Plus Protein Dual Xtra Standards (Bio-Rad Laboratories GmbH, Feldkirchen, Germany) were used as a molecular weight marker. Proteins were transferred to a nitrocellulose membrane (Bio-Rad Laboratories GmbH, Feldkirchen, Germany) on a semidry transfer apparatus (Trans-Blot Turbo, Bio-Rad Laboratories GmbH, Feldkirchen, Germany). Membranes were blocked for 1 h at room temperature with 5% skim dry milk (Sigma) in TBS-Tween 0.1% (TBS-T), and subsequently probed with rabbit anti-NPC1 antibody (abcam, 1:500) and mouse anti-beta-actin (abcam, 1:10,000) in 3% skim dry milk in TBS-Tween 0.1% (TBS-T) overnight at 4 °C. Membranes were washed with TBS-T and incubated with fluorescence-coupled secondary antibodies (goat anti-rabbit IgG (H&L) Antibody DyLight™ 680 and goat anti-mouse IgG (H&L) Antibody DyLight™ 800); both Rockland Immunochemicals Inc., Limerick, PA, USA), 1:10.000 in 3% nonfat dry milk in TBS-T, for 2 h at room temperature. Bands were detected and visualized using the Odyssey Infrared Imaging System (LI-COR Biosciences GmbH, Bad Homburg vor der Höhe, Germany). Densitometry of the resulting bands was performed in ImageStudioLite. The signals were normalized to those obtained for beta-actin.

### 4.11. Endoglycosidase H Assay

For the cleavage of N-linked glycans, 20 µg of non-denatured protein samples were incubated with denaturing buffer (1 µL) at 37 °C for 10 min. The reaction was subsequently treated with Glycobuffer (2 µL), ddH20 (7 µL), and Endo H (1000 U, 1 µL, New England Biolabs GmbH, Frankfurt am Main, Germany) and incubated at 37 °C for 2 h. The deglycosylated protein was separated by SDS-PAGE and immunodetected as described above.

### 4.12. Statistical Analyses

Statistical significance was assessed by the unpaired student′s *t*-test. Statistics were performed using the software GraphPad Prism 6 (GraphPad Software Inc., San Diego, CA, USA). *p*-values < 0.05 were considered to indicate significant differences. Samples of at least three independent experiments were quantified and the results are presented as mean ± SD. * = *p* < 0.05, ** = *p* < 0.01, *** = *p* < 0.001.

## 5. Conclusions

We present in vitro data and the clinical presentation of a female NP-C1 patient, carrying the compound heterozygous mutation p.V1023Sfs*15/p.G992R. The p.G992R mutation is described to be linked with a “variant” biochemical phenotype and an adult disease onset. The V1023Sfs*15 had not been described yet and thus it was of interest to report the cellular phenotype as well as the disease course of the patient. Based on the results of the in vitro profile of patient-specific fibroblasts and in addition to iPSC-derived neural differentiated cells and hepatocyte-like cells, we classified the observed biochemical phenotype as “variant”. In accordance with other case reports about the p.G992R mutation in combination with other mutations, the patient′s disease course reflects an adult disease onset with a severe and, in part, fast progression of the disease. This, once more, advises that an early diagnosis is crucial for a timely pharmacological intervention, in order to slow down the course of the disease, improve the patient′s quality of life, and extend life expectancy.

## Figures and Tables

**Table 1:** Clinical findings at the point in time of the diagnosis of NP-C1.

	Unit	Patient	References
creatinkinase	U/l	17	30–179
triglyceride	mg/dL	152	<150
cholesterol (total)	mg/dL	216	<200
HDL-cholesterol	mg/dL	40	>40
LDL/HDL		3.6	<3
iron	µg/dL	39	50–170
transferrin	%	8.7	15–45
erythrocytes	/pl	4.91	3.7–4.8
chitotriosidase	nmol/mL/h	203	20–100

## Data Availability

The data presented in this study are available on request from the corresponding author.

## Abbreviations

αFP	Alpha-Fetoprotein
ALB	Albumin
BSA	Bovine Serum Albumin
ChT	Chitotriosidase
DE	Definitive Endoderm
DMSO	Dimethyl sulfoxide
Endo H	Endoglycosidase H
ER	Endoplasmic reticulum
GCS	Glucosylceramide synthase
GFAP	Glial fibrillary acidic protein
HDL	High Density Lipoprotein
HLCs	Hepatocyte-like cells
HNF4α	Hepatocyte nuclear factor 4 alpha
HPCs	Hepatic progenitor cells
ICG	Indocyanine green
iPSCs	Induced pluripotent stem cells
LDL	Low Density Lipoprotein
LSM	Laser Scanning Microscope
LSO	Lysosome-like storage organelle
NDCs	Neural differentiated cells
NPCs	Neural progenitor cells
NPC1	Niemann-Pick type C1 protein
NP-C1	Niemann-Pick disease type C1
PAS	Periodic acid-Schiff
PFA	Paraformaldehyde
PSA-NCAM	Polysialylated-neural cell adhesion molecule
